# Contrasting Root and Photosynthesis Traits in a Large-Acreage Canadian Durum Variety and Its Distant Parent of Algerian Origin for Assembling Drought/Heat Tolerance Attributes

**DOI:** 10.3389/fchem.2017.00121

**Published:** 2017-12-14

**Authors:** Paula Ashe, Hamid Shaterian, Leonid Akhov, Manoj Kulkarni, Gopalan Selvaraj

**Affiliations:** National Research Council of Canada, Saskatoon, SK, Canada

**Keywords:** abiotic stress, drought, durum wheat, heat, photosynthesis, root

## Abstract

In Canada, the world's top exporter of high-protein durum, varietal development over its nearly six-decade history has been driven by a quest for yield improvement without compromise on grain protein content and other quality aspects. Pelissier, a landrace selection from Algeria that was introduced into North America more than a century ago and the variety Strongfield that was released in 2004 are notable. Pelissier, known to elaborate more roots and considered as drought tolerant, has been cultivated commercially and thus deemed adapted. Strongfield has Pelissier in its pedigree, and it remains a high-acreage variety. Strongfield was found to elaborate only about half of the root biomass of Pelissier at maturity in greenhouse trials under well-watered conditions. Extended drought stress caused a significant reduction in the root biomass of both lines. However, Pelissier under drought maintained at least as much root biomass as that of Strongfield under well-watered conditions. In comparison to Pelissier, it had a superior photosynthesis rate (27.16 μmol CO_2_ m^−2^ s^−1^), capacity for carboxylation (V_cmax_: 132.83 μmol CO_2_ m^−2^ s^−1^) and electron transport/ribulose-1,5–bisphosphate (RuBP) regeneration (J_max_: 265.40 μmol CO_2_ m^−2^ s^−1^); the corresponding values for Pelissier were 19.62 μmol CO_2_ m^−2^ s^−1^, 91.87 μmol CO_2_ m^−2^ s^−1^, and 163.83 μmol CO_2_ m^−2^ s^−1^, respectively, under well-watered conditions. Under short-term/mild drought conditions, the carbon assimilation rate remained stable in Pelissier while it declined in Strongfield to the Pelissier level. However, Strongfield succumbed to extended drought sooner than Pelissier. Photosynthesis in Strongfield but not Pelissier was found to be sensitive to high temperature stress. These results provide encouraging prospects for further exploitation of beneficial physiological traits from Pelissier in constructing climate-resilient, agronomically favorable wheat ideotypes.

## Introduction

The rate of yield increase in wheat is ~0.9% and it is insufficient to double the food production that is required to feed a burgeoning global population (Reynolds et al., 2012; Ray et al., 2013). Yield reduction due to environmental stresses such as drought and heat worsens the problem (Daryanto et al., 2016). Climate change models project increased warming and alterations in precipitation extremes (Maloney et al., 2014). Rising temperature will cause greater loss in wheat productivity in regions that are already low-yielding (Asseng et al., 2017). The estimated average loss is 6.0 ± 2.9% per degree Celsius (Zhao et al., 2017). In Canada, numerous years of widespread moisture deficit have been reported (Marchildon et al., 2008, 2009) and “an impending water crisis” in western Canada, where much of the country's wheat is produced, has also been predicted (Schindler and Donahue, 2006). Factors such as these will greatly impact yield and food security (Curtis and Halford, 2014; Yusa et al., 2015).

Durum wheat, which originated in the Eastern Mediterranean region and is cultivated in rainfed regions of the world that are prone to drought and heat stress, is considered to be less sensitive to abiotic stress than bread wheat (Monneveux et al., 2012). However, a recent study indicates that durum performs poorly under stressful conditions when compared with bread wheat (Marti and Slafer, 2014), indicating that addition of drought tolerance traits to durum is important. Global consumption of durum flour products such as pasta and couscous has risen and over 14 million tons of pasta were produced in 2014 (http://www.internationalpasta.org/resources/World%20Pasta%20Industry%20Survey/IPOstatreport2014low.pdf). Canada contributes approximately 40% of world durum export (http://atlas.media.mit.edu/en/profile/sitc/0411/), and nearly all of it is cultivated in the semi-arid regions of the prairies. The history of durum cultivation and breeding in Canada is relatively recent, and ongoing varietal development is under stringent requirement for maintenance of grain quality, yield and agronomy. Initially, varieties from other countries such as the USA, Russia, Algeria and South Africa were grown (Knott, 1995; Dexter, 2008; McCallum and Depauw, 2008; Clarke et al., 2010). Pelissier, a century-old Algerian selection from a landrace (Clark et al., 1922) that offered much superior gluten strength, was brought in via the northern plains of the US to supply grain for pasta production (Dexter, 2008). Pelissier, however, had a high lipoxygenase content that broke down the yellow pigment (Knott, 1995) and it was supplanted by other varieties yielding yellow flour, a feature that was preferred in pasta. Durum accumulates cadmium in the grains (Zook et al., 1970; Hart et al., 1998), raising concerns as the export markets became sensitive to potential toxic effects of cadmium in their food (EFSA, 2009). Strongfield was registered in 2004 (Clarke et al., 2005) as the first Canadian durum cultivar with a low grain cadmium content while retaining the high protein trait, better flour color and excellent agronomic characteristics (Dexter, 2008). Within 2 years, it became the largest commercial durum variety in Western Canada (Canadian Wheat Board statistics via Canadian Grain Commission) and retained this position through 2015, and in 2016 the combined acreage of Strongfield with another closely related variety was at 46% of the 4.32 million acres (http://www.grainscanada.gc.ca/statistics-statistiques/variety-variete/varieties-en.htm). Many current varieties also include Strongfield in their pedigree, thus highlighting the importance of this variety.

In terms of developing drought tolerant wheat for Canadian agriculture, there were early efforts, starting with the work of Aamodt and coworkers in the 1930s (Aamodt, 1935; Aamodt and Johnston, 1936). Pelissier had been included in these comparisons and was identified as one elaborating more roots than drought sensitive lines. Hurd (1964) confirmed the robust root system of Pelissier and used it as a parent to introgress the trait in varietal development (Hurd et al., 1972, 1973). Field studies demonstrated that under drought stress Pelissier had the lowest reduction in yield and the lowest drought susceptibility index among the genotypes assessed (Gebeyehou and Knott, 1983). Pelissier is known to retain leaf water content which is also a contributing factor to its drought tolerance (Dedio, 1975; Clarke and McCaig, 1982). Hurd (1974) pointed out that to breed for drought resistance breeders should select, in addition to extensive roots systems especially at lower soil depth, sustainable photosynthesis under stress.

Sustained photosynthesis is central to plant growth, development and productivity. Water stress can diminish photosynthesis in a number of ways: for example, reduction in the leaf area; stomatal closure; ultrastructural changes to chloroplasts; lower leaf water content; poor electron transport and photosynthetic reactions (Dubey, 2005). High photosynthetic efficiency is important for yield and research is underway to achieve this in wheat (Parry et al., 2011) and the prospects are optimistic (Long et al., 2006; Driever et al., 2014). Enhanced photosynthetic efficiency could also prove to be of greater importance under stress conditions (Araus et al., 2002; Reynolds et al., 2016). For example, Reynolds et al. (2000) and Gutiérrez-Rodríguez et al. (2000) demonstrated a positive correlation between yield and higher photosynthetic rate in warm environmental conditions. Cultivars that are able to maintain photosynthetic capacity under stress conditions are an important addition to breeding programs.

Strongfield is a typical representative of modern durum cultivars of premium quality grain and it is considered well adapted to the prairies (Bueckert and Clarke, 2013). However, there has been no information on the physiological and genetic underpinnings in Strongfield that contribute to its performance nor additional physiological traits that would be beneficial for productivity under drought and heat stress. The publically available whole genome sequence assembly of Strongfield (https://wheat-urgi.versailles.inra.fr/Seq-Repository/Assemblies) can be utilized effectively by genomics-based approaches only if the phenotypic attributes are also characterized. The objective of this study is to compare the root traits and photosynthesis efficiency of Strongfield with Pelissier toward a goal of deploying genomics technologies to derive superior combinations of stress tolerance traits. Pelissier is particularly well suited for such a comparison: It is a drought tolerant variety that has indeed been cultivated to a significant extent till the late 1980s in Canada and it is a distant parent with multiple occurrences in the pedigree of Strongfield (McCallum and Depauw, 2008).

## Materials and methods

### Varieties

Strongfield and Pelissier seeds were kindly provided by Agriculture and Agri-Food Canada, Swift Current, Saskatchewan, Canada.

### Plant growth–greenhouse

We had compared development of the root system of these two varieties in many ways. We used field soil or soil-less growth mix in standard 15 cm pots and plastic tubes, and custom-made acrylic slabs of various dimensions and eventually chose to use Sunshine Mix #8 (Sun Gro Horticulture, AB, Canada) in PVC tubes (120 × 7.5 cm; root tubes) and acrylic slabs (2.5 × 20 × 40 cm; referred to as root boxes or slabs) in order to have a better control over applying a suitable drought regimen and to better analyze root biomass. The slab faces were covered to prevent light exposure to the roots. This soil-less media will be referred to as “soil” and it was supplemented with 2 grams of Osmocote 14-14-14 slow release fertilizer in slabs and 4 grams in tubes. Plants were grown in a greenhouse at 25/22°C (day/night) and 16 h/8 h (light/dark) with natural lighting supplemented when levels reached <300 μmol m^−2^ s^−1^. When plants reached booting (Zadoks stage 45) half of the plants were subjected to drought stress (target of 25% holding capacity). Control (well-watered) plants were maintained at a target of 95% holding capacity. All plants were weighed and watered twice per week to the target levels of water holding capacity with the exceptions noted in Results.

### Plant growth–field

Seeds were sown in single row plots in three locations: Agriculture and Agri-Food Canada fields in Saskatoon, Saskatchewan (N 52°9′, W 106° 34′; Site 1, 2016), AgQuest Saskatoon (52° 7′ 41″, W 106° 50′ 52″; Site 2, 2016) and AgQuest Saskatoon (N 52° 7′ 20.1″, W 106° 50′ 13.7″; Site 3, 2017). Fourteen rows of each line were planted and weeded as required throughout the growing season. Growing degree days (GDD) from seedling emergence date as the start point was derived from ((T_max_+T_min_)/2) − T_base_ where T_max_ and T_min_ represent the maximum and minimum temperature reached each day and T_base_ as 10°C.

### Photosynthesis measurements

Carbon assimilation was measured using an LI-6800 photosynthesis system (LI-COR Inc., Lincoln, NB). The chamber was set to 400 ppm CO_2_ and a light intensity of 1,000 μmol m^−2^ s^−1^. Single point measurements were performed on a minimum of 4 plants per treatment per line in the greenhouse and on all 14 replicates of each line in the field at two time points. In early experiments, to determine the response to drought, the measurements were made 14–21 days after drought onset (Zadoks stage 64–69). Well-watered plants were assessed at matching stages on each day of measurements. In the time- course experiments, plants were maintained at 95% holding capacity until booting (Zadoks stage 45) at which point plants slated for drought stress were left to reach the new target of 25% holding capacity; watering was continued in the well-watered slabs to maintain holding capacity at 95%. On Day 0, all plants were weighed and watered to ensure calibration at the appropriate initial holding capacity. Control measurements (Day 0) were performed on plants at their target holding capacity, 2–4 h post watering. Measurements were on alternate days with watering being withheld until the endpoint was reached. The endpoint was determined by assessment of plant health and water status.

The effect of heat in the greenhouse was assessed by setting the block temperature on the LI-6800 to 25°C (control) or 38° (heat stress) and equilibrating leaves for 20 min at this temperature. This resulted in flag leaf temperatures of 25 and 35°C respectively as the leaves stabilized below the set block temperature for heat stress. Measurements were made on 4 plants per variety for each water status. These were repeated on two independent days for confirmation; the results from the first replication are shown.

Light curves were conducted under control (25°C) and heat stressed (32 and 35°C; leaf temperature) conditions in well-watered plants to determine the effect on maximum photosynthetic assimilation (A_max_); each time point was held for 90 to 180 s to achieve stability, CO_2_ was set to 400 ppm and light intensities were increased incrementally from 0 to 2,500 μmol m^−2^ s^−1^. Curves from 4 plants of each variety were analyzed using the R script provided by Heberling (2013). CO_2_ curves were conducted under control (25°C well-watered) conditions with light intensity of 1,500 μmol m^−2^ s^−1^; each step was held for 90 to 240 s for stability. V_cmax_ and J_max_ values were determined from 3 plants of each variety using the R package Plantecophys (Duursma, 2015).

### Field heat stress conditions

Site 1: No-stress photosynthesis measurements refer to data on July 15th, 2016 (111.6 mm total precipitation, 411 GDD) when the average maximum temperature was 23.5°C over the preceding 7 days; photosynthesis under stress was assayed on July 26th, 2016 (121.4 mm total precipitation, 512 GDD) after the preceding 7 days had an average maximum temperature of 27.3°C. Fourteen plots per variety were measured on each date. Site 2: Measurements were performed on July 29th, 2016 (115.7 mm total precipitation and 491 GDD). The mean maximum temperature 7 days prior to measurements was 26.9°C. Site 3: Measurements were performed on July 14th, 2017 (42.6 mm total precipitation and 390 GDD). The average maximum temperature 7 days prior to these measurements was 26.8°C. Measurements were repeated on July 31st, 2017 (60 mm total precipitation and 567 GDD) with an average maximum temperature of 29.0°C the 7 days prior to data collection. Note, the comparison was for a given location and not across because of differences in GDD and temperature.

### Root biomass

Three weeks following the onset of drought (following the collection of all physiological data) the roots were scanned to visualize architecture then were harvested to determine total root biomass. The roots were washed with water and rinsed thoroughly to remove growing media. They were dried for 24 h at 65°C then weighed using a Mettler AE163 balance.

### Data analysis

One and two-way ANOVA and *t*-tests were performed using GraphPad Prism software version 6 (La Jolla, CA). CO_2_ and light curve data was analyzed using the R package Plantecophys (Duursma, 2015) or the R script made available by Heberling (2013).

## Results

### Strongfield lacks the robust root trait of its ancestor pelissier

**Figure 1:**
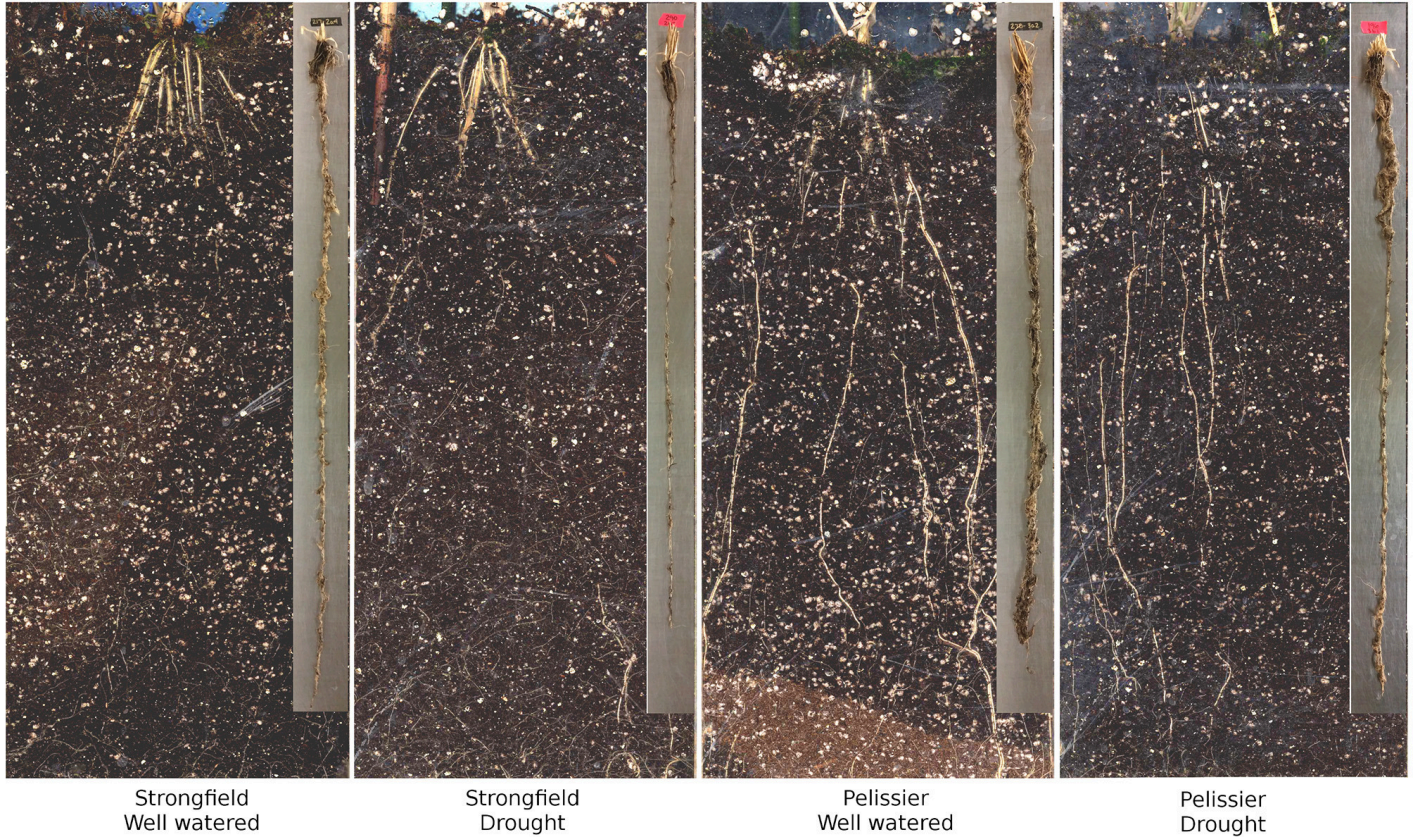
Root distribution of Strongfield and Pelissier under well-watered and drought stress conditions in root boxes as described in Methods. The original has been digitally enhanced for brightness and contrast. Retrieved and washed roots of Strongfield and Pelissier are shown in the inset.

**Figure 2:**
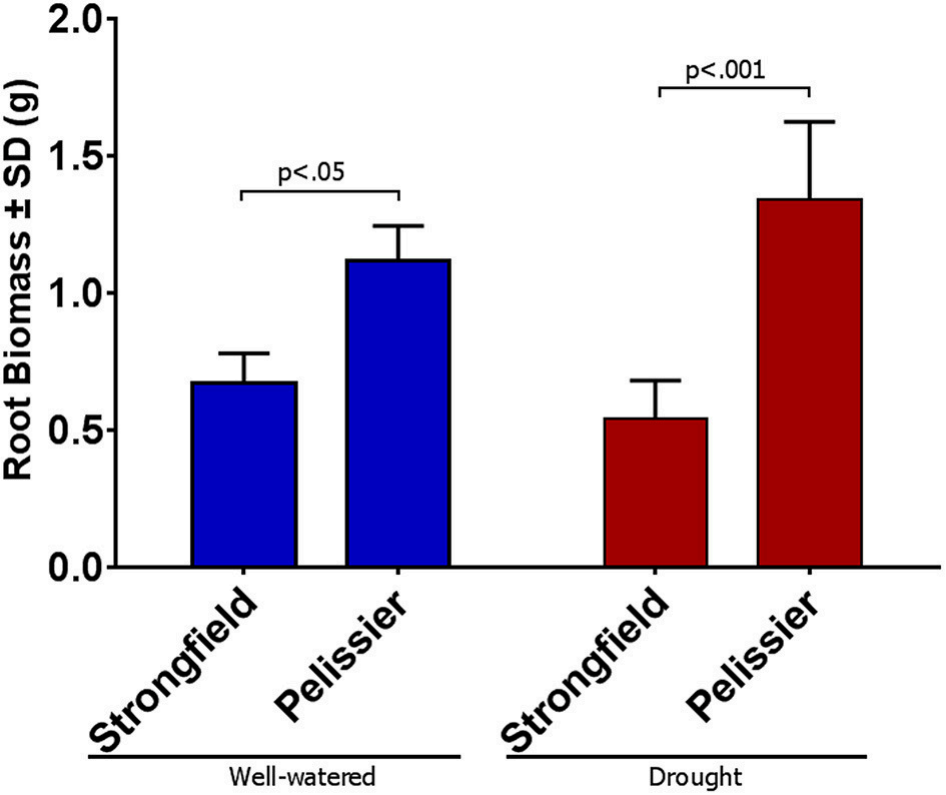
Root biomass per plant in Strongfield and Pelissier plants at grain filling stage on dry weight basis. Roots from 4 plants of each line in each condition were harvested in grain filling period from root boxes 3 weeks after the water holding capacity reached 25% (drought); the well-watered controls were maintained at 95% capacity.

We assessed the root phenotype in plants in their grain-filling stages or in fully senesced harvest-ready plants. While the absolute values for the root biomass expressed on a per plant basis varied between trials done at different seasons or between root tubes and root boxes, the contrast between the two lines was consistent within a given trial in all of these various iterations: Strongfield produced significantly less root biomass than Pelissier, regardless of the environmental conditions or the growth container used (P. Ashe et al., unpublished results). A representative experiment to illustrate the difference between the two lines in root boxes is shown (Figures 1, 2). The dry weight of Strongfield root at 0.67 g per plant was about half of the Pelissier root under watered and drought conditions over 3 weeks. The differences were significant in two-way ANOVA [main effect of variety *F*_(1, 11)_ = 52.36, *p* < 0.0001]. Under these milder drought conditions over 3 weeks, the impact of drought on root biomass *within* a given line was less obvious. Both lines lost significant root biomass upon prolonged drought to maturity. However, such droughted Pelissier was still able to maintain a root biomass greater than or equal to the root biomass value of well-watered Strongfield since it had nearly twice as much root biomass to start with prior to drought onset. These results establish that the robust root biomass trait of Pelissier had not been selected for in Strongfield during the course of breeding.

### Strongfield shows superior carbon dioxide assimilation in unstressed conditions, but pelissier retains photosynthetic capacity under extended drought

**Figure 3:**
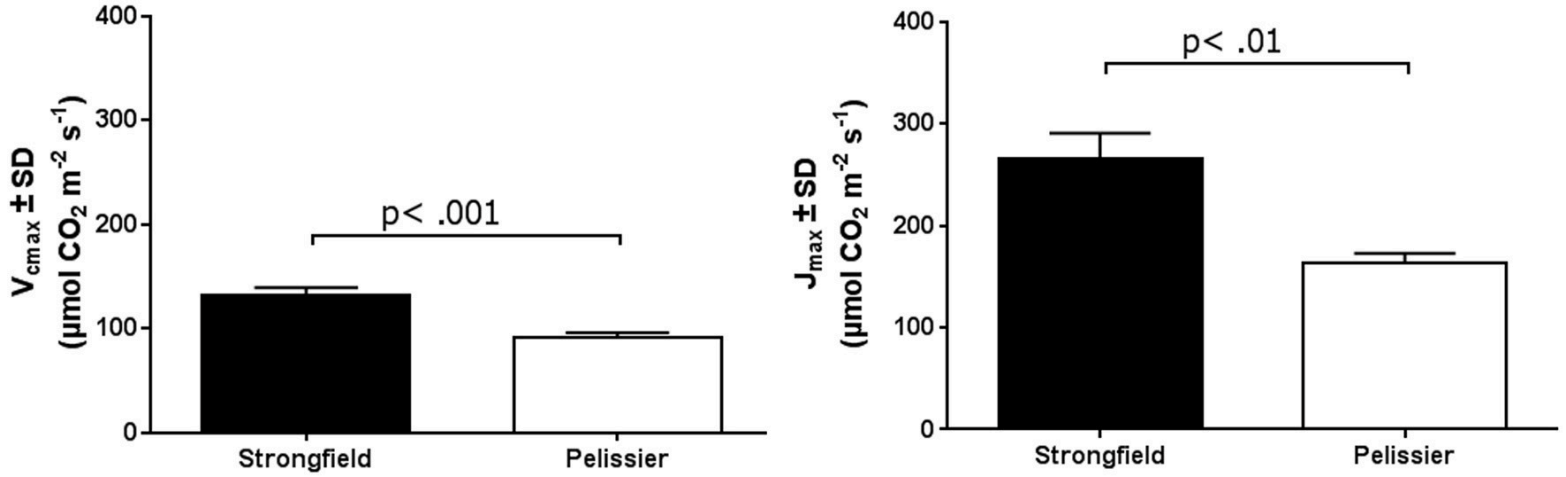
V_cmax_ and J_max_ of Strongfield and Pelissier as determined from CO_2_ curves of 3 well-watered plants per variety.

**Figure 4:**
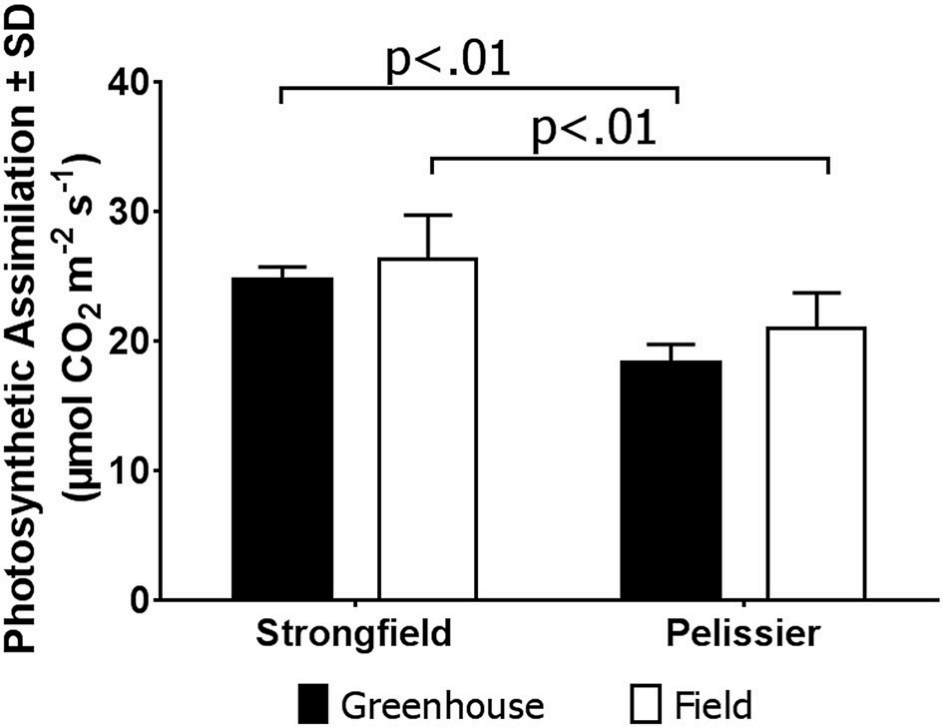
Carbon assimilation rate in Strongfield and Pelissier plants. The flag leaf of well watered plants in greenhouse and plants without any obvious symptoms of moisture stress in the field was assayed. A minimum of 4 plants per line per environment were assessed.

**Figure 5:**
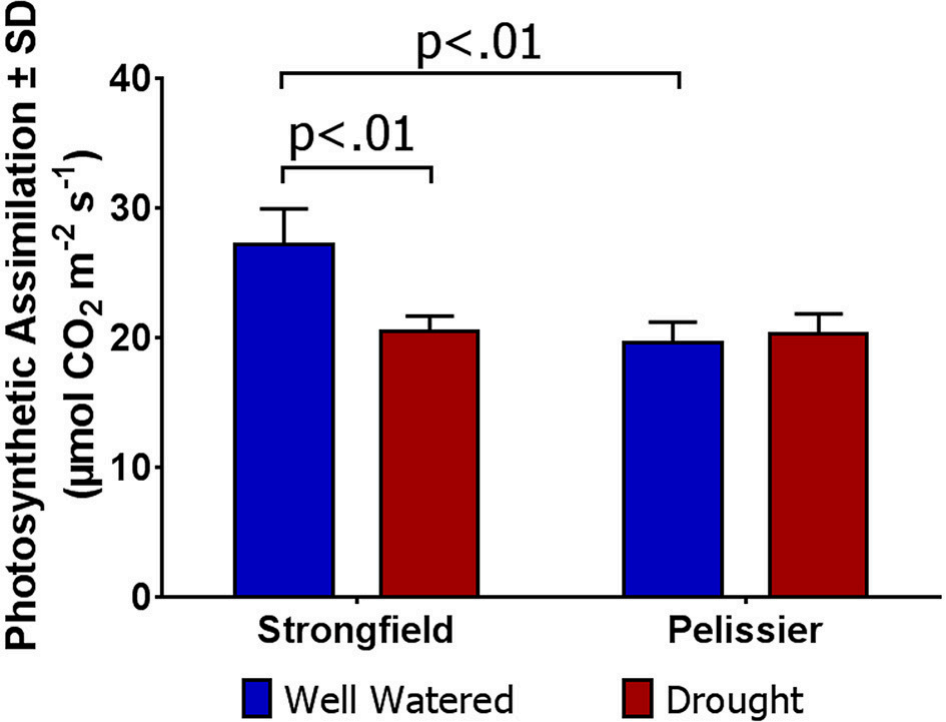
Carbon assimilation rate in Strongfield and Pelissier subjected to drought stress. Drought was imposed on plants grown in root tubes in a greenhouse.

As shown in Figure 3, measurements of photosynthesis on the flag leaf of Strongfield and Pelissier demonstrated significantly higher levels of carboxylation efficiency (V_cmax_) and electron transport rate (ETR)/enzymatic reactions involved in regenerating ribulose-1,5–bisphosphate (RuBP) for the Calvin-Benson cycle to operate. Strongfield had a V_cmax_ of 132.83 μmol CO_2_ m^−2^ s^−1^ and a J_max_ of 265.40 μmol CO_2_ m^−2^ s^−1^. These values were significantly greater than the V_cmax_ of Pelissier at 91.87 μmol CO_2_ m^−2^ s^−1^ (unpaired *t*-test, *t* = 8.780, df = 4, *p* = 0.0009) and J_max_ at 163.83 μmol CO_2_ m^−2^ s^−1^ (unpaired *t*-test, *t* = 6.551, df = 4, *p* = 0.0028). Generally, both V_cmax_ and J_max_ scale together indicating coordination of the underlying processes. The J_max/_V_cmax_ ratios were not significantly different (2.00 ± 0.12 and 1.79 ± 0.15 in Strongfield and Pelissier, respectively). The CO_2_ assimilation rates were higher in Strongfield under the well-watered conditions of a greenhouse or field environment without overt moisture stress (Figure 4). Two-way ANOVA showed a significant effect of variety [*F*_(1, 18)_ = 31.28; *p* < 0.0001] but no significant effect of environment. Under water stress conditions, however, Strongfield showed a significant reduction in CO_2_ assimilation rate whereas notably, Pelissier remained stable over 3 weeks of drought (Figure 5). Two-way ANOVA shows a significant interaction between treatment and variety [*F*_(1, 9)_ = 12.80; *p* < 0.01]. Under these drought stress conditions imposed in a greenhouse, the assimilation rates in Strongfield and Pelissier were similar.

**Figure 6:**
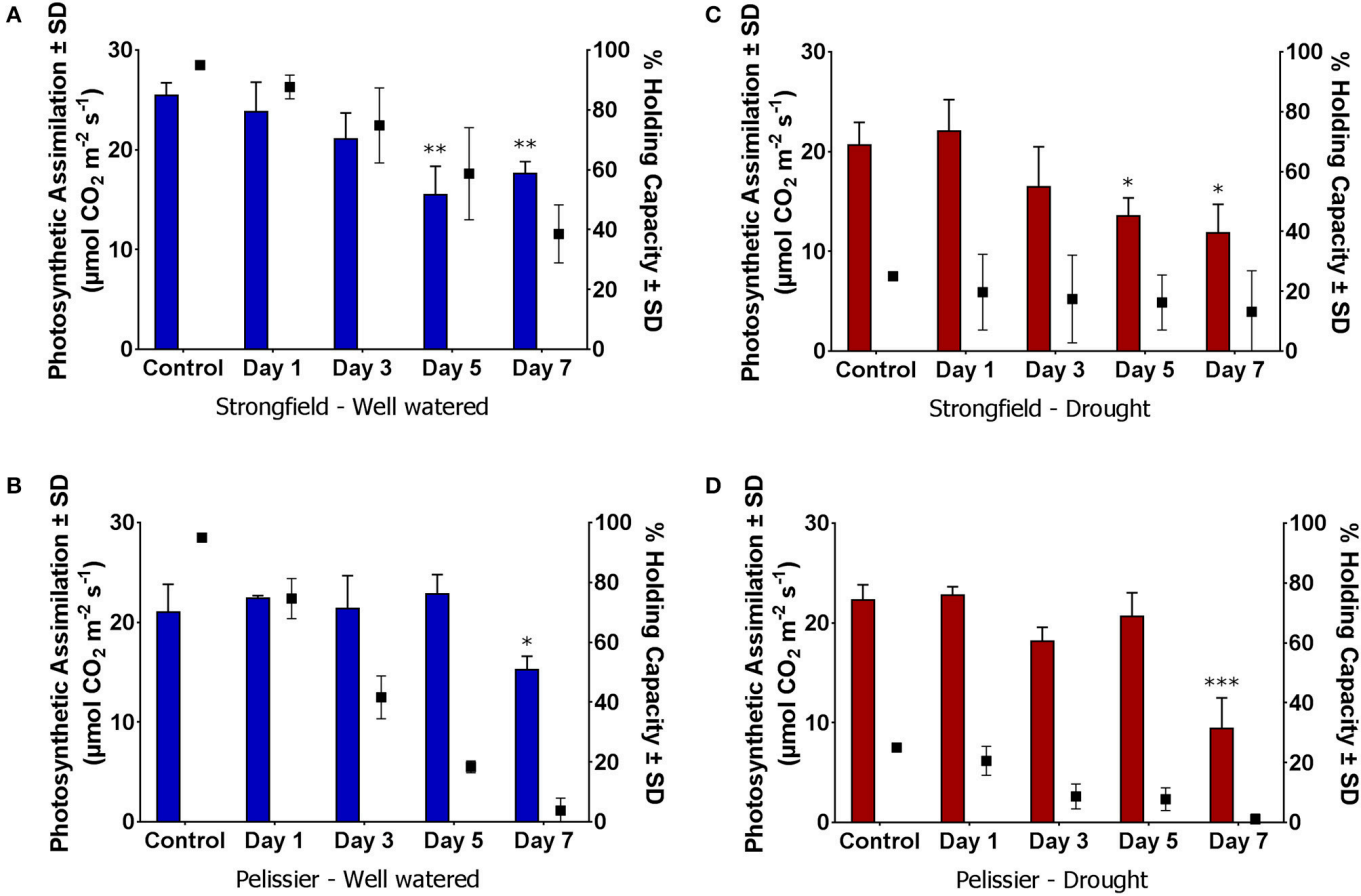
Reduction in photosynthetic CO_2_ assimilation upon gradual moisture loss from initially water replete and droughted conditions. **(A,C)** Initial water holding capacity at 95%; **(B,D)** Initial water holding capacity 25%; “Control” refers to measurements taken within 2–4 h of watering on Day 0. Day 1 through 7, no watering. Water availability data is summarized in Table 1 and depicted here for ease of visualization of changes in photosynthesis with reductions in available water. Water holding capacity shown on right y-axis (∙ symbol). One-way ANOVA followed by Dunnett's multiple comparisons test was performed for each variety under each water status treatment (^*^p < 0.05, ^**^p < 0.01, ^***^p < 0.001).

To discern further differences between the two varieties, each was subjected to moisture deficit from two starting points. The plants had been maintained at 95% capacity by regular watering until the initiation of booting (Zadoks stage 45). From this baseline, watering was either continued to maintain 95% (well-watered) or discontinued. The latter reached approximately 25% holding capacity after 5 days at which point re-watering was done to ensure all plants were at 25%. From this Day-0, watering was withheld for 7 days in all cases. Pelissier withdrew water at a faster rate than Strongfield and nearly depleted it by Day 7 even though both lines did not vary in their average tiller number of 3 (Table 1). In contrast, nearly half of the initial water supply remained in the Strongfield boxes at Day 7. At Day 5, for instance, 69 to 80% of the initial “soil water” content had been taken up from droughted and well-watered starting points of Pelissier while only 35 to 39% had been withdrawn by Strongfield. The evaporative loss was negligible because of the small surface-to-volume ratio of the root boxes. The CO_2_ assimilation rate in Strongfield declined significantly at Day 5 when the “soil water” was at nearly 60% from a starting point of 95% (Figure 6A). In contrast, the photosynthesis rate in Pelissier was stable till Day 5 even when the soil water had declined to 18%; this line showed a significant reduction in its CO_2_ assimilation only when the soil water content declined to <4% (Figure 6C), indicating stability of its photosynthesis in drought conditions. Similar insensitivity of Pelissier to extended drought was evident when soil water declined from an initial value of 25 to 8% (Figure 6D) that contrasted with the sensitivity of Strongfield that became evident at 16% (Figure 6B). Note that at Day 7, Pelissier plants had almost no soil water left when the decline in photosynthesis rate became evident.

**Table 1:** Water content remaining in root boxes after withholding water.

	**Day 1**	**Day 3**	**Day 5**	**Day 7**
Strongfield – 95%	87.65 ± 3.95	74.80 ± 12.53	58.70 ± 15.39	38.55 ± 9.69
Strongfield – 25%	19.68 ± 12.65	17.40 ± 14.65	16.25 ± 9.17	13.13 ± 13.70
Pelissier – 95%	74.60 ± 6.70	41.60 ± 7.16	18.37 ± 1.94	3.78 ± 4.13
Pelissier – 25%	20.57 ± 4.84	8.67 ± 4.15	7.77 ± 3.80	1.03 ± 1.79

*The starting water content in the root boxes was 95% holding capacity for well-watered, and 25% for droughted plants. These water contents were maintained by weighing the boxes and watering the plants as needed. For this particular set of experiments, watering was withheld for 7 days in all cases and the water loss noted in the table, expressed as percent holding capacity (Mean ± SD), is reported in the text after normalization to the two initial water contents*.

### Carbon dioxide assimilation under heat stress in field conditions is superior in pelissier

**Figure 7:**
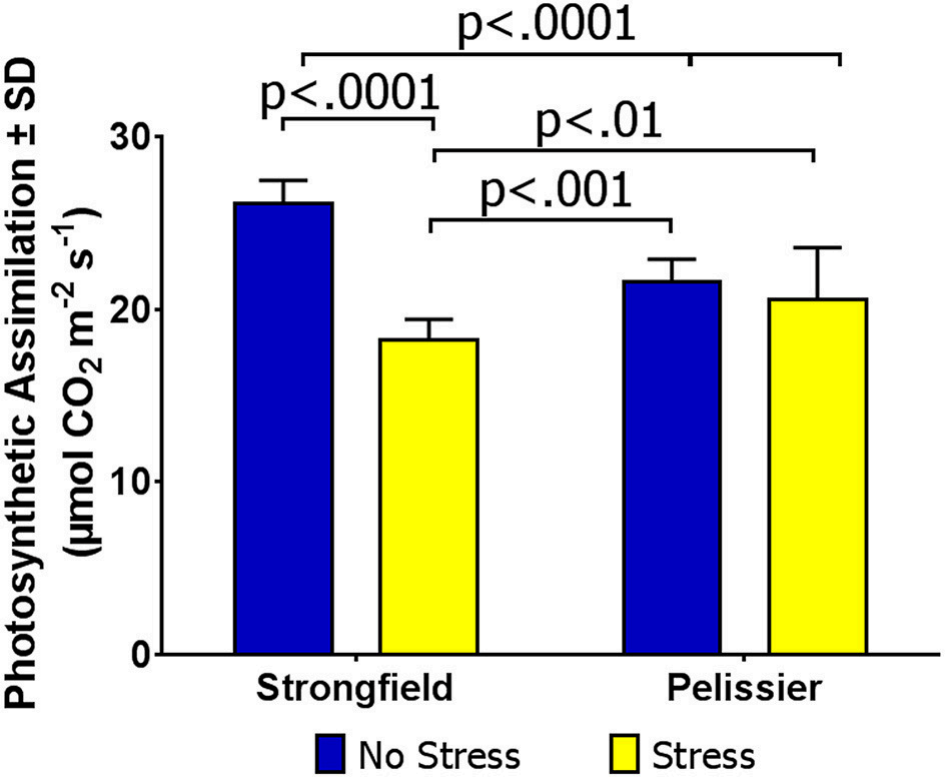
Carbon assimilation rate in Strongfield and Pelissier under field conditions of stress and no-stress.

Under the environmental conditions in rented commercial farms without irrigation, drought stress was inconsistent and crops were at times exposed to periods of intense heat with or without accompanying drought. Taking advantage of such occurrences, as detailed in Methods, we assayed field-grown plants for photosynthesis under “no-stress” and stress conditions. Figure 7 shows the results of Site 1 where the measured leaf temperature was 25.1°C for the “no-stress” condition and 27.2°C for the stress condition. Two-way ANOVA showed a significant interaction between treatment and variety [*F*_(1, 51)_ = 46.73; *p* < 0.0001] and inspection of this interaction indicated that Strongfield was sensitive to stress while Pelissier was stable for photosynthesis. It should be noted that while the CO_2_ assimilation rate in Pelissier was insensitive to heat stress and significantly higher than Strongfield under heat stress, the pre-heat stress rate was lower when compared with Strongfield. At Site 2, following a 7-day period of high temperature, CO_2_ assimilation rate when the leaf temperature was 27.2°C showed similar photosynthetic rate in Strongfield (17.84 μmol CO_2_ m^−2^ s^−1^ ± 1.43) and Pelissier (mean carbon assimilation = 18.79 μmol CO_2_ m^−2^ s^−1^ ± 0.88). The trials at Site 3 in 2017 where the average leaf temperature under stress conditions was 29.7°C compared to 26.0°C under no-stress conditions also confirmed that photosynthesis in Pelissier was tolerant to heat stress (no-stress: 23.6 μmol CO_2_ m^−2^ s^−1^ ± 1.42; under stress = 25.2 μmol CO_2_ m^−2^ s^−1^ ± 2.74) while it was sensitive in Strongfield (no-stress = 28.7 μmol CO_2_ m^−2^ s^−1^ ± 2.42; under stress = 21.8 μmol CO_2_ m^−2^ s^−1^ ± 2.49). Additional experiments outlined below also supported this conclusion.

**Figure 8:**
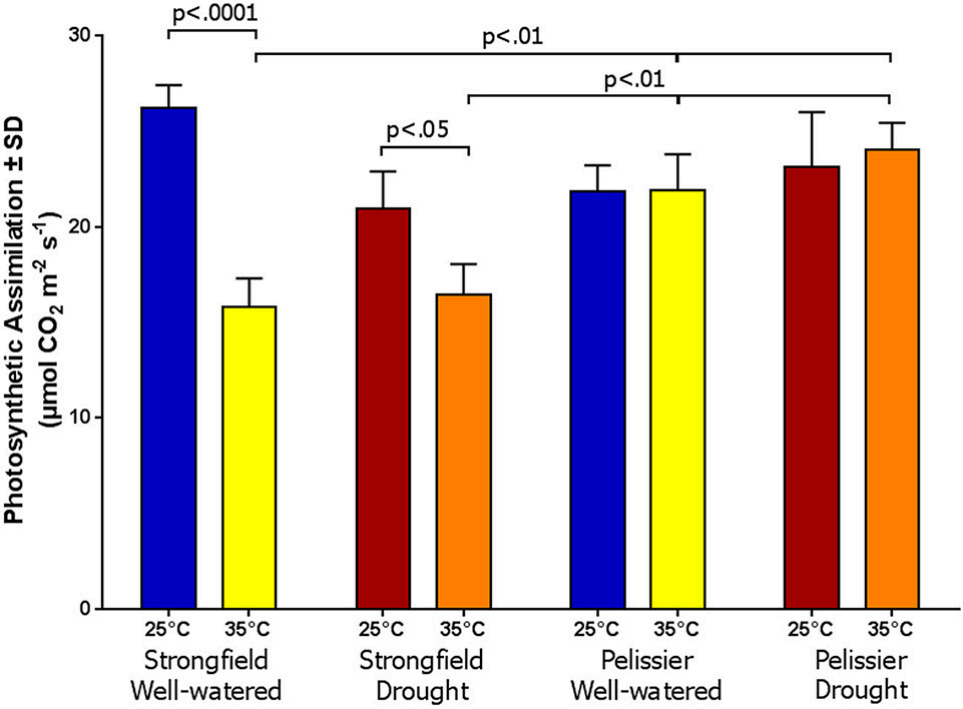
Photosynthesis is Strongfield and Pelissier at two leaf temperatures and watering conditions.

**Figure 9:**
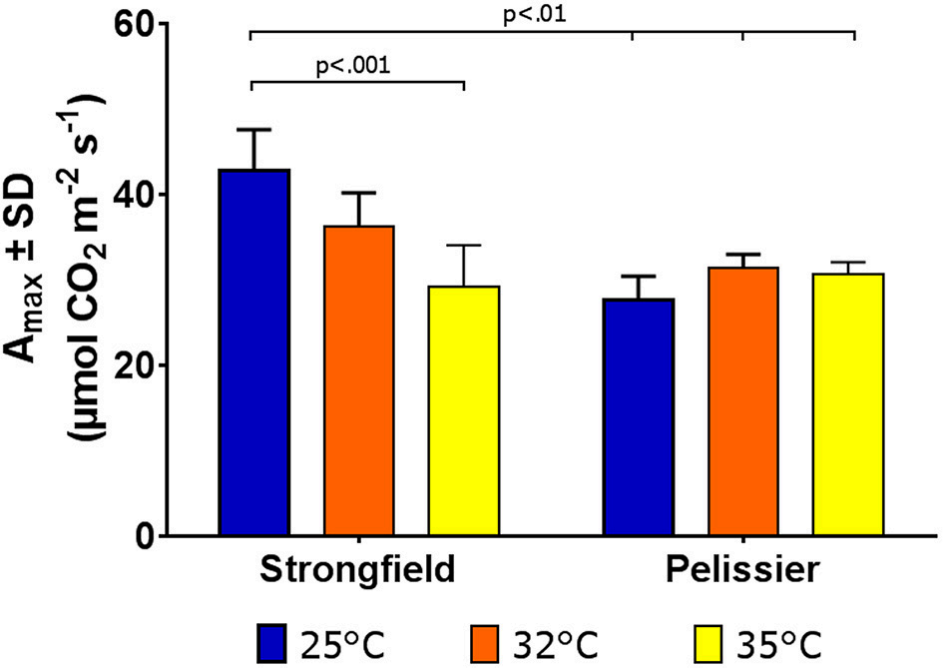
Maximum photosynthetic capacity in Strongfield and Pelissier. A_max_ was determined from light curves on flag leaf under well-watered conditions.

Greenhouse-grown plants were assessed for photosynthesis at two different flag leaf temperatures by altering the temperature in the measuring chamber of LI-COR. Photosynthesis was measured following a 20 min incubation period to stabilize the leaf temperature (Figure 8). Here, both well-watered plants (95% holding capacity) and droughted plants (25% holding capacity) were investigated. Strongfield showed a significant reduction in carbon assimilation following heat stress under both well-watered and drought stress conditions; heat stress depressed the assimilation rate more severely than drought alone did, and imposing drought and heat stress did not further reduce the assimilation rate. Pelissier remained stable irrespective of the drought and temperature status in these experiments. Two-way ANOVA of the data in Figure 8 showed a main effect of both variety [*F*_(3, 19)_ = 9.031; *p* = 0.0006] and temperature [*F*_(1, 19)_ = 24.04; *p* < 0.0001] and a significant interaction [*F*_(3, 19)_ = 11.71; *p* = 0.0001]. Fast light curves were performed to determine maximum photosynthetic assimilation (A_max_) in well-watered plants. Strongfield had a significantly higher A_max_ than Pelissier at 25°C, which decreased at 32 and 35°C (Figure 9). Two-way ANOVA showed a main effect of both variety [*F*_(1, 16)_ = 16.37 *p* = 0.0009] and temperature [*F*_(2, 16)_ = 4.719; *p* = 0.0245] and a significant interaction [*F*_(2, 16)_ = 11.02; *p* = 0.0010]. The maximum capacity for photosynthesis under ambient CO_2_ levels in the atmosphere was greater in Strongfield than in Pelissier. However, Pelissier remained stable; at 35°C both lines had comparable A_max_.

## Discussion

Our study reveals that Strongfield, a variety that has the most cumulative acreage in Canadian durum cultivation history, produces much less root biomass relative to its ancestor Pelissier and it succumbs to drought more readily than Pelissier. The yield data in Clarke et al. (2005) show that Strongfield suffered substantial reduction in 2001 and 2002 relative to 2000 in the western and southwestern prairies. This region experienced severe drought in 2001 and 2002 (Bonsal and Regier, 2007; Wheaton et al., 2008). This suggests that improving drought tolerance in modern cultivars will be beneficial. This situation is not unlike independent breeding programs elsewhere, for example in China, where the predecessors of modern cultivars are more drought tolerant (Sun et al., 2014). Terminal drought exacts a toll on wheat grain yield (Farooq et al., 2014). While breeders generally do not consider selection for root traits because of the practical difficulties, Rich et al. (2016) have recently found that many wheat varieties selected for yield in rainfed dry regions where the crop relies on water stored in deep soil at grain filling period indeed show deep rooting traits. Root biomass in subsoil (below 20 cm) correlates positively with grain yield in wheat under drought stress (Fang et al., 2017). Even small amounts of water absorbed from subsoil at depths of 135 to 185 cm contribute to grain yield such that 10 mm of water extracted translates to 0.62 t ha^−1^ (Kirkegaard et al., 2007). It is known that drought resistant and susceptible varieties differ in water flow rate and hydraulic conductivity (Emam and Bijanzadeh, 2012). As Vadez (2014) points out, hydraulics underpinned by structural, physiological and biochemical aspects should also be considered for improving drought tolerance. While these aspects remain to be studied, our work confirms the robust root phenotype of Pelissier (Hurd, 1964) as an opportunity to introgress the trait into modern cultivars. In spite of the presence of Pelissier in the genealogy of Strongfield, the root trait of Pelissier had not been selected for. As with any individually superior traits, circumventing associated linkage drag, if present, will be essential. In European wheat, linkage drag has indeed played a role in the exclusion of higher root biomass (Voss-Fels et al., 2017).

It is a formidable task to double food crop output in the next three decades. At the current rate of wheat yield increase, there would be a 30% shortage by 2050 (Ray et al., 2013). Kromdijk and Long (2016) dub the situation as “one crop breeding cycle from possible starvation” in view of the time required for developing varieties to balance this deficit. Much of the present yield gain in wheat is attributed to enhanced harvest index that has very little room, if any, for improvement because further reduction in aerial biomass will be counterproductive. Physiological processes present challenges as traits for selection in crop breeding. Best physiological trait lines are known to yield more than their best parent (Reynolds and Langridge, 2016). Combining better photosynthetic capacity with a suitable root system for optimal water extraction should improve productivity under abiotic stress. The photosynthesis parameters reported here indicate that uptake, carboxylation and ETR (and/or RuBP regeneration) are superior in Strongfield under well-watered conditions. However, CO_2_ assimilation is sensitive to drought albeit not lower than the level seen in Pelissier under short term drought. This suggests that the greater capacity of Strongfield (A_max_) for photosynthesis and the higher rate of photosynthesis likely contribute to its overall productivity, making it a widely adapted variety (Bueckert and Clarke, 2013). Pelissier, however, tolerates severe and extended drought to which Strongfield succumbs, indicating that Pelissier harbors useful traits that have not been introgressed into Strongfield. The drought stress stability of photosynthesis is an intriguing aspect to investigate in further studies. Also interesting is the heat-insensitive photosynthetic CO_2_ assimilation in Pelissier in contrast to the significant reduction in Strongfield. It might be beneficial if stress stability is combined with the higher photosynthesis rate of Strongfield. Rubisco requires a functional activase (Rca) and there are two Rca isoforms (Salvucci et al., 1987). Whereas, Rubisco activation by Rca is sensitive to heat, a recent study shows that inhibition of Rca, rather than the quantity of it, in heat-stressed wheat impacts photosynthesis (Perdomo et al., 2017). This aspect remains to be explored.

As Mahon (1983) points out, four basic criteria must be satisfied for a physiological trait to be incorporated in breeding: (1) genetic variability; (2) knowledge of genetic control; (3) agronomic benefit; and (4) measurability in large-scale trials. Encouragingly, advancements in exploiting photosynthetic rate are being achieved that build on genetic variability and understanding the genetic controls (Evans, 2013; Lin et al., 2015; Prins et al., 2016; Carmo-Silva et al., 2017). The genetic and biochemical bases of the root and stress-stable photosynthesis characteristics need to be unraveled to render modern varieties drought/heat stress tolerant. Any arising physiological adjustments and their impact on yield should also be investigated. Breeders endeavor to avoid undesirable carry-over traits that impact varietal development. This restraint in genetic crosses is particularly evident in the recent history of wheat breeding in Canada. The short growing season and stringent agronomic and grain quality parameters for varietal registration limit the options for sourcing genetic diversity. Pelissier as a variety that has been commercially grown in Canada is especially suitable as a donor of greater root biomass and stress stability of photosynthesis to improve upon the beneficial attributes of Strongfield and other similar modern durum varieties. A biparental population of Strongfield and Pelissier from Agriculture and Agri-Food Canada is being mapped (M. Kulkarni et al., unpublished results), further aided by the availability of a wheat whole genome sequence (http://www.wheatgenome.org/News/Latest-news/RefSeq-v1.0-URGI) and an assembly of Strongfield (https://urgi.versailles.inra.fr/download/iwgsc/TGAC_WGS_assemblies_of_other_wheat_species) and transcriptomics of roots and flag leaf that is underway in our laboratory. The mechanistic basis of the stress stability in Pelissier and that of the higher efficiency of photosynthesis in Strongfield under non-stress conditions, when elucidated, will assist in constructing suitable ideotypes for evaluation of beneficial combinations of physiological traits and for deployment in varieties.

## Author contributions

HS, LA, MK, and PA: Performed experiments. PA and GS: Wrote the MS that was read and approved by all authors.

### Conflict of interest statement

The authors declare that the research was conducted in the absence of any commercial or financial relationships that could be construed as a potential conflict of interest.
